# Adjuvant treatment with Wu-Zi-Yan-Zong formula for abnormal sperm parameters associated with male infertility: a meta-analysis of randomized controlled trials

**DOI:** 10.3389/fphar.2025.1580705

**Published:** 2025-05-06

**Authors:** Feilun Cui, Yueshi Zhang, Yu Fan

**Affiliations:** ^1^ Department of Urology, Affiliated Taizhou Second People’s Hospital of Yangzhou University, Taizhou, China; ^2^ Department of Urology, The Fourth Affiliated Hospital of Jiangsu University, Zhenjiang, China; ^3^ Department of Medical Laboratory Science, Liaoning University of Traditional Chinese Medicine, Shenyang, Liaoning, China; ^4^ Cancer Institute, The Affiliated People’s Hospital, Jiangsu University, Zhenjiang, China

**Keywords:** Wu-Zi-Yan-Zong formula, abnormal sperm parameters, male infertility, randomized controlled trials, meta-analysis

## Abstract

**Background:**

Wu-Zi-Yan-Zong (WZYZ) formula is a traditional Chinese botanical drug that has been used to treat male infertility. This meta-analysis aims to evaluate its effectiveness of the WZYZ formula as an adjuvant therapy for treating abnormal sperm parameters associated with male infertility.

**Methods:**

A comprehensive literature search was conducted using PubMed, Embase, Cochrane Library, Web of Science, SinoMed, Wanfang, and CNKI databases until December 12, 2024 to identify randomized controlled trials (RCTs) that assessed the effectiveness of the WZYZ formula as an adjuvant therapy for treating abnormal sperm parameters associated in men with infertility. For dichotomous data, the pooled results were summarized as risk ratio (RR) with 95% confidence intervals (CI), while continuous data were expressed as pooled weighted mean difference (WMD) with 95% CI.

**Results:**

A total of 11 RCTs involving 951 men were identified. The pooled results showed that the WZYZ formula, when combined with control treatment, significantly improved the pregnancy rate of female partners (RR 1.68; 95% CI 1.34–2.11), semen volume (WMD 0.58 mL; 95% CI 0.28–0.89), sperm concentration (WMD 6.87 × 10^6^/mL; 95%CI 4.24–9.51), total sperm motility (WMD 15.55%; 95% CI 10.38–20.72), forward grade (a) sperm motility (WMD 5.44%; 95% CI 1.86–9.01), forward grade (a + b) sperm motility (WMD 7.14%; 95% CI 4.04–10.23), abnormal sperm morphology (WMD −10.38%; 95% CI −15.72 to −5.03), and activity of the acrosome enzyme (WMD 8.02 × 10^6^ μIU; 95% CI 3.58–12.46.

**Conclusion:**

Adjuvant treatment with WZYZ formula significantly improves the pregnancy rate of female partners by improving several semen parameters in infertile men with abnormal sperm parameters. However, further well-designed RCTs with larger sample sizes are necessary to definitively determine the efficacy and safety of the WZYZ formula in treating abnormal sperm parameters associated with male infertility.

**Systematic review registration:**

https://www.crd.york.ac.uk/PROSPERO/view/CRD42024629510.

## 1 Introduction

Male infertility is a major challenge in reproductive health, affecting approximately 8%–12% of couples of reproductive age worldwide (Agarwal et al., 2021). Male factors alone account for 30%–50% of these cases (Eisenberg et al., 2023). Common causes of male infertility include low sperm count, abnormal sperm function and quality, and obstruction of the reproductive tract. Varicoceles, which can lead to spermatogenesis failure, are also a frequent cause of male infertility (Cho and Seo, 2014). Abnormal sperm parameters can present as issues with azoospermia, oligospermia, asthenozoospermia, teratozoospermia, sperm DNA damage, or a combination of these phenotypes. Various treatments, such as antioxidants, aromatase inhibitors, clomiphene, L-carnitine, coenzyme Q10, vitamin E, and tamoxifen, have been shown to improve semen parameters and sperm quality (Rambhatla et al., 2024). However, despite significant advancements in medical care, the effectiveness of pharmacological therapies and varicocele repair in achieving successful pregnancy remains unsatisfactory (Kim et al., 2016; Sharma et al., 2022). Currently, there is a lack of specific and effective medications for treating abnormal sperm parameters associated with male infertility.

Traditional Chinese Medicine (TCM) has been widely used in the treatment of male infertility (Zhou et al., 2019). The Wu-Zi-Yan-Zong (WZYZ) formula, a classical Chinese botanical drug, was firstly documented in the “She Sheng Zhong Miao Fang”. Its primary therapeutic effect is nourishing the kidneys and producing essence, making it commonly used to treat conditions such as premature ejaculation, erectile dysfunction, seminal emission, and infertility (Tai et al., 2024). Studies have shown that the WZYZ formula, whether used alone or in combination with biomedicine, has demonstrated promising benefits for male infertility by improving sperm parameters, including sperm concentration, viability, motility, and morphology (Chen et al., 2002). However, an early meta-analysis of five randomized controlled trials (RCTs) suggested that the evidence for the WZYZ formula’s effectiveness was limited due to the small number of trials and methodological flaws in the included studies (Zhao et al., 2018). A more recent meta-analysis published in Chinese language indicated that the WZYZ formula, either alone or in conjunction with biomedicine, was superior to biomedicine alone in improving sperm concentration, motility, and the conjugal pregnancy rate (Duan and Ma, 2024). However, this well-designed meta-analysis did not comprehensively review the literature, and several RCTs (Cui et al., 2011; Luo et al., 2011; Wu et al., 2013; Suo et al., 2017; Li et al., 2018; Bai et al., 2020; Su et al., 2020) were excluded from the analysis. Therefore, there is still a lack of definitive evidence regarding the therapeutic efficacy of the WZYZ formula in treating male infertility. The objective of this meta-analysis is to evaluate the effectiveness of the WZYZ formula as an adjuvant therapy for treating abnormal sperm parameters associated with male infertility, based on the most current available RCTs.

## 2 Materials and methods

### 2.1 WZYZ formula composition and taxonomic validation

The WZYZ formula is a composition of five different plants: *Cuscuta chinensis Lam* (Convolvulaceae; *Semen Cuscutae), Lycium barbarum L* (Solanaceae; *lycii fructus*), *Rubus idaeus L* (Rosaceae; *Rubi Fructus*), *Schisandra chinensis (Turcz.) Baill* (Schisandraceae; *Chinese magnolia-vine*), and *Plantago asiatica L*. (Plantago; *Plantain seed*). These plants are combined in a relative proportion of 35%:35%:17%:5%:8%. The composition of the WZYZ formula has been validated taxonomically using resources such as the Medicinal Plant Names Services (MPNS) and Plants of the World Online (POWO). The WZYZ pill and capsule have been authorized by the China Drug and Food Administration and are prepared through a series of processes, including weighing, crushing, sieving, mixing, pelletizing, drying, and packaging. Each sachet weighs either 6 g or 9 g.

### 2.2 Data sources and search strategy

To conduct this meta-analysis, we followed the Preferred Reporting Items for Systematic Reviews and Meta-Analyses (PRISMA) guidelines (Moher et al., 2009). The protocol for this meta-analysis was prospectively registered with PROSPERO (CRD42024629510). A comprehensive literature search was conducted across international databases (PubMed, Embase, Cochrane Library, and Web of Science) as well as Chinese databases (SinoMed, Wanfang, and CNKI) until December 12, 2024, without any language restrictions. The following combined terms and keywords were used for the literature search: (“WuziYanzong” OR “Wu zi Yan zong”) AND (“male infertility” OR (“sperm” OR “oligospermia” OR “asthenospermia” OR “oligoasthenospermia”) AND (“randomized controlled trial” OR “random” OR “randomized”). A detailed account of the search strategy can be found in Supplementary Material S1. Additionally, we manually reviewed the reference lists of the included trials and related reviews to identify any potentially missing articles.

### 2.3 Study selection

Two independent reviewers screened the titles and abstracts, subsequently selecting eligible trials based on the PICOS criteria: 1) Patients: adult men diagnosed with infertility due to azoospermia, oligospermia, asthenozoospermia, teratozoospermia, sperm DNA damage, or a combination of these conditions; 2) Intervention: WZYZ formula (in the form of a pill, capsule, or decoction) combined with biomedicine or surgery; 3) Comparison: Biomedicine or surgery alone as the control intervention; 4) Outcomes: the pregnancy rate of female partners as the primary endpoint, and semen volume, sperm concentration, total sperm motility, forward grade (a) sperm motility, forward grade (a + b) sperm motility, abnormal sperm morphology, and acrosome enzyme activity as secondary endpoints; and 5) RCTs published in peer-reviewed journals. The abnormal sperm parameters considered were sperm concentration <20 × 10^6^/mL, total sperm motility <60%, forward grade (a) sperm motility <25%, forward grade (a + b) sperm motility <50%, abnormal morphology >96%, sperm DNA damage, and low acrosome enzyme activity. The exclusion criteria included: 1) the WZYZ formula was not used as an adjuvant therapy; 2) the WZYZ formula was combined with other Chinese botanical preparations or acupuncture as an intervention; 3) a modified WZYZ formula was used as an intervention; and 4) non-RCTs (retrospective studies or self-controlled trials).

### 2.4 Data extraction and risk of bias assessment

Two reviewers independently abstracted the following data into a pre-designed table: first author’s name, publication year, country of origin, etiology of infertility, sample sizes, patients’ ages, form of the WZYZ formula, WZYZ regimen, control intervention, treatment duration, semen parameters, and pregnancy status of female partners. The risk of bias in the included trials was assessed using the Cochrane Risk of Bias Tool for RCTs, which evaluates random sequence generation, allocation concealment, blinding, incomplete outcome data, selective outcome reporting, and other biases, including the patient selection process considering syndrome differentiation in TCM. Each item was categorized as “low risk,” “high risk,” or “unclear risk.”. Two independent reviewers examined the retrieved full-text articles, extracted the aforementioned data, and assessed the risk of bias. Any discrepancies between the two reviewers were resolved by consensus with a third reviewer.

### 2.5 Statistical analysis

Stata software version 12.0 (StataCorp, College Station, TX) and RevMan version 5.2 (Oxford, United Kingdom) were used for the meta-analysis. For dichotomous data, the pooled results were summarized as risk ratio (RR) with 95% confidence intervals (CI). Continuous data were expressed as pooled weighted mean difference (WMD) with 95% CI. To assess heterogeneity among the trials, the Cochrane Q statistic and *I* ^2^ index were utilized. A Cochrane Q test result of less than 0.10 and an *I* ^2^ index greater than 50% indicate the presence of significant heterogeneity. In cases of significant heterogeneity, a random effects model was applied for the meta-analysis; otherwise, a fixed-effects model was used. A leave-one-out sensitivity analysis was conducted to evaluate its impact on the overall pooled results. Subgroup analyses were performed based on the preparation of the WZYZ formula, etiology of infertility (varicocele/obstructive azoospermia or idiopathic), and whether grouping was done according to TCM syndrome differentiation. Publication bias was assessed using Begg’s test (Begg and Mazumdar, 1994) and Egger’s test (Egger et al., 1997). The trim-and-fill method was used to adjust the pooled results when publication bias was detected. GRADE analysis was employed to evaluate the certainty of evidence, which involved assessing the risk of bias, inconsistency, indirectness, imprecision, and publication bias. The level of evidence for each outcome was categorized as high quality, moderate quality, low quality, or very low quality.

## 3 Results

### 3.1 Search results

**FIGURE 1:**
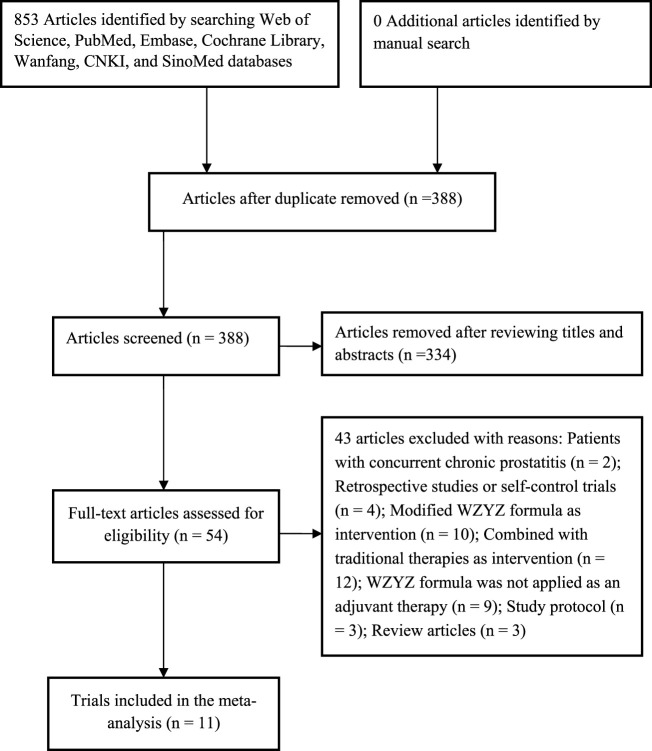
Flow chart of the trial selection process.

A total of 853 articles were obtained using the aforementioned search strategy. After excluding 465 duplicate records, 334 articles were reviewed for relevance based on their titles and abstracts. Of these, 334 were found to be irrelevant and were removed, leaving 54 articles for full-text eligibility assessment. After applying the inclusion and exclusion criteria, 43 articles were further excluded. The primary reason for exclusion was that they used a modified WZYZ formula or combined other traditional therapies as interventions, rather than solely using the WZYZ formula as an adjuvant therapy. Ultimately, 11 RCTs (Li et al., 2009; Zhang et al., 2010; Cui et al., 2011; Luo et al., 2011; Zhu, 2011; Wu et al., 2013; Suo et al., 2017; Li et al., 2018; Bai et al., 2020; Huang et al., 2020; Su et al., 2020) were included in this meta-analysis (Figure 1).

### 3.2 Characteristics of the included studies

**FIGURE 2:**
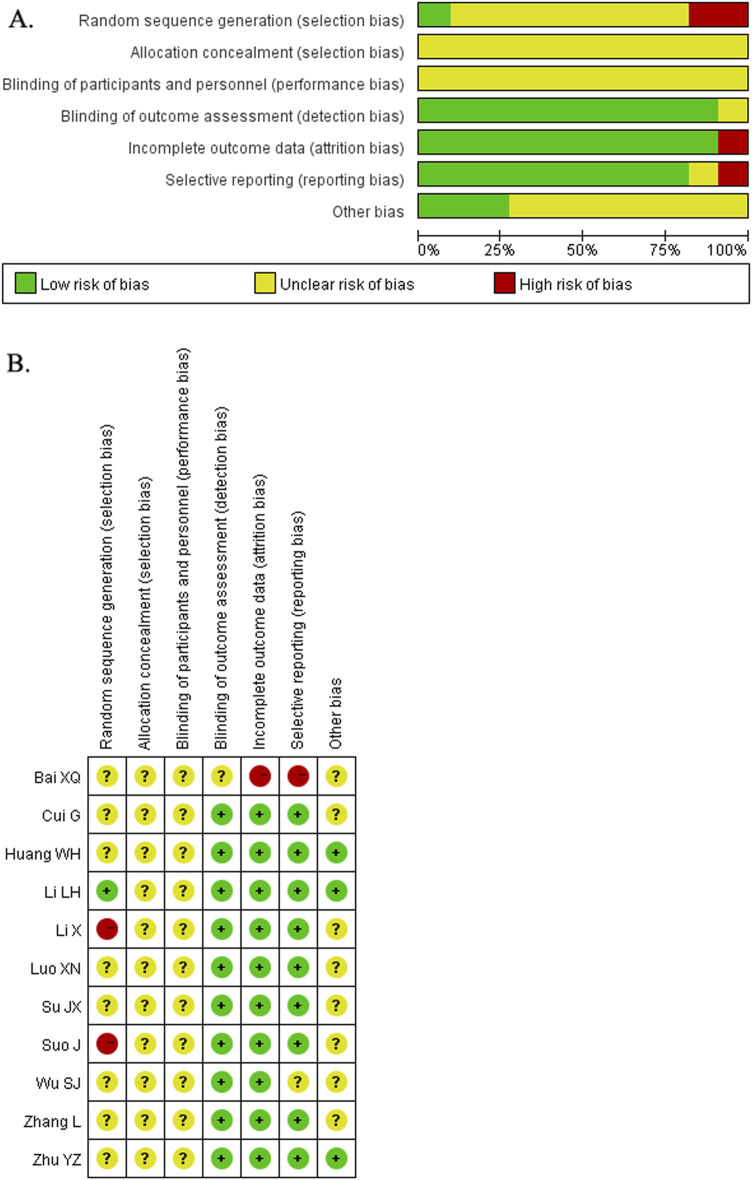
Risk of bias graph **(A)** and Risk of bias summary **(B)**.

The characteristics of the included RCTs are summarized in Table 1. All eligible RCTs were conducted in China and published between 2009 and 2020. Three RCTs (Zhu, 2011; Li et al., 2018; Huang et al., 2020) enrolled patients based on TCM syndrome differentiation, while the remaining trials did not specify this criterion. A total of 951 men participated in the included RCTs, with sample sizes ranging from 32 to 120. Among the included RCTs, two (Luo et al., 2011; Wu et al., 2013) employed surgical interventions, while the others utilized pharmacological treatments, including L-carnitine, Vitamin E, Vitamin C, selenium yeast, clomiphene, prednisone, or testosterone undecanoate. L-carnitine was the most frequently prescribed medication. The preparations of the WZYZ formula used included pills, capsules, and decoctions. The duration of the interventions varied from 12 weeks to 3 months. Although all included RCTs reported randomization, only one trial (Li et al., 2018) provided adequate details on sequence generation. Allocation concealment and blinding were not mentioned in any of the included RCTs. Two RCTs (Zhang et al., 2010; Luo et al., 2011) reported dropouts or withdrawals. Overall, the included RCTs were assessed to have an unclear risk of bias (Figure 2).

**TABLE 1:** Main characteristics of the included trials.

Author/year	Patients	Sample sizes	Age (years)	Infertility duration (years)	WZYZ group	Control group	Treatment course	Outcome measures
Li et al. (2009)	Asthenospermia	WZYZ:71 Con:46	WZYZ:22–40 Con:23.5–39	—	WZYZ pill + Testosterone undecylate	Testosterone undecylate	3 months	②+③+④+⑤+⑧
Zhang et al. (2010)	Oligospermia, asthenospermia	WZYZ:26 Con:29	28.6 ± 0.5	3.5 ± 0.2	WZYZ pill + L-carnitine + Vitamin E	L-carnitine + Vitamin E	3 months	①+②+③+④+⑤+⑧
Cui et al. (2011)	Oligospermia, asthenospermia	WZYZ:50 Con:50	24–41	—	WZYZ decoction + L-carnitine	L-carnitine	3 months	①+②+③+④+⑤
Luo et al. (2011)	Obstructive azoospermia	WZYZ:16 Con:16	24–38	—	WZYZ pill + Transurethral resection of ejaculatory duct	Transurethral resection of ejaculatory duct	7 months	①+④+⑤+⑧
Zhu (2011)	Infertility	WZYZ:50 Con:50	34.8 ± 8.1	4.1 ± 1.1	WZYZ pill + Clomiphene + Prednisone	Clomiphene + Prednisone	3 months	①+③+⑥+⑧
Wu et al. (2013)	Varicocele	WZYZ:29 Con:29	WZYZ:27.5 ± 1.6 Con:28.5 ± 1.4	—	WZYZ pill + Ligation spermatic Vein + Vitamin C	Ligation spermatic Vein + Vitamin C	3 months	①+⑤+⑦+⑧
Suo et al. (2017)	Asthenospermia	WZYZ:58 Con:58	WZYZ:31.7 ± 2.6 Con:31.5 ± 2.8	WZYZ:3.3 ± 1.5 Con:3.1 ± 1.6	WZYZ capsule + L-carnitine	L-carnitine	3 months	①+②+③+④+⑤+⑧
Li et al. (2018)	Oligospermia, asthenospermia	WZYZ:60 Con:60	WZYZ:29.6 ± 3.3 Con: 30.1 ± 4.6	WZYZ:1–6 Con:1–6.5	WZYZ capsule + L-carnitine	L-carnitine	3 months	①+②+③+④+⑥+⑧
Su et al. (2020)	Oligospermia, asthenospermia	WZYZ:52 Con:51	WZYZ:27.3 ± 5.3 Con:29.1 ± 4.6	WZYZ:1.7 ± 2.6 Con:1.9 ± 2.3	WZYZ capsule + Vitamin E	Vitamin E	3 months	①+②+③+⑤+⑥+⑧
Bai et al. (2020)	Acrosomal enzyme deficiency	WZYZ:40 Con:40	WZYZ:30.6 ± 5.1 Con:31.2 ± 5.7	WZYZ:1–10 Con:1–11	WZYZ decoction + L-carnitine	L-carnitine	3 months	③+④+⑤+⑦
Huang et al. (2020)	Asthenospermia	WZYZ:35 Con:35	23–46	—	WZYZ pill + Vitamin E + Selenium yeast	Vitamin E + Selenium yeast	12 weeks	②+④+⑤+⑧

WZYZ, Wu-Zi-Yan-Zong; Con, control; ①, Semen volume; ②, Sperm concentration; ③, Total sperm motility; ④, Forward sperm motility (grade a); ⑤, Forward sperm motility (grade a + b); ⑥, Percentage of abnormal morphology; ⑦, Activity of acrosome enzyme; ⑧, Pregnancy rate of female partners.

### 3.3 Pregnancy rate of female partners

**FIGURE 3:**
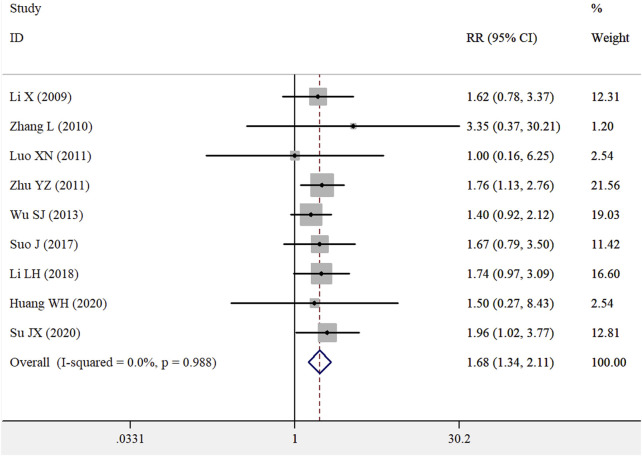
Pooled RR with 95% CI for pregnancy rates of female partners compared with or without Wu-Zi-Yan-Zong formula treatment.

Nine RCTs (Li et al., 2009; Zhang et al., 2010; Luo et al., 2011; Zhu, 2011; Wu et al., 2013; Suo et al., 2017; Huang et al., 2020; Li et al., 2020; Su et al., 2020) were conducted to investigate the effect of the WZYZ formula as an adjuvant therapy on the pregnancy rate of female partners. The results showed that the pregnancy rate in the group receiving the WZYZ formula combined with the control therapy was 35.0%, compared to 20.9% in the control therapy group. As illustrated in Figure 3, the pooled RR of pregnancy rate for WZYZ formula plus control therapy was 1.68 (95% CI 1.34–2.11) compared to control therapy alone, as determined by a fixed-effect model. No significant heterogeneity was observed among these trials (*I* ^2^ = 0.0%; *p* = 0.988). A leave-one-out sensitivity analysis showed that no individual trial significantly influenced the overall pooled risk estimate (Supplementary Figure S1). The WZYZ formula as an adjuvant therapy significantly improved pregnancy rate in all subgroups except for the varicocele/obstructive azoospermia subgroup (Supplementary Table S1). Both Begg’s test (*p* = 1.000) and Egger’s test (*p* = 0.436) showed no evidence of publication bias.

### 3.4 Semen volume

Six RCTs (Zhang et al., 2010; Cui et al., 2011; Zhu, 2011; Suo et al., 2017; Li et al., 2018; Su et al., 2020) examined the effects of the WZYZ formula as an adjuvant therapy on semen volume. As illustrated in Supplementary Figure S2, the pooled WMD in the semen volume was 0.58 mL (95% CI 0.28–0.89) for the WZYZ formula combined with control therapy versus control therapy alone, as determined by a random effects model. However, significant heterogeneity was observed among the included trials (*I* ^2^ = 84.1%; *p* < 0.001). A leave-one-out sensitivity analysis was conducted to confirm the robustness of the original pooled effect sizes. Additionally, Begg’s test (*p* = 0.260) and Egger’s test (*p* = 0.322) indicated no evidence of publication bias.

### 3.5 Sperm concentration

**FIGURE 4:**
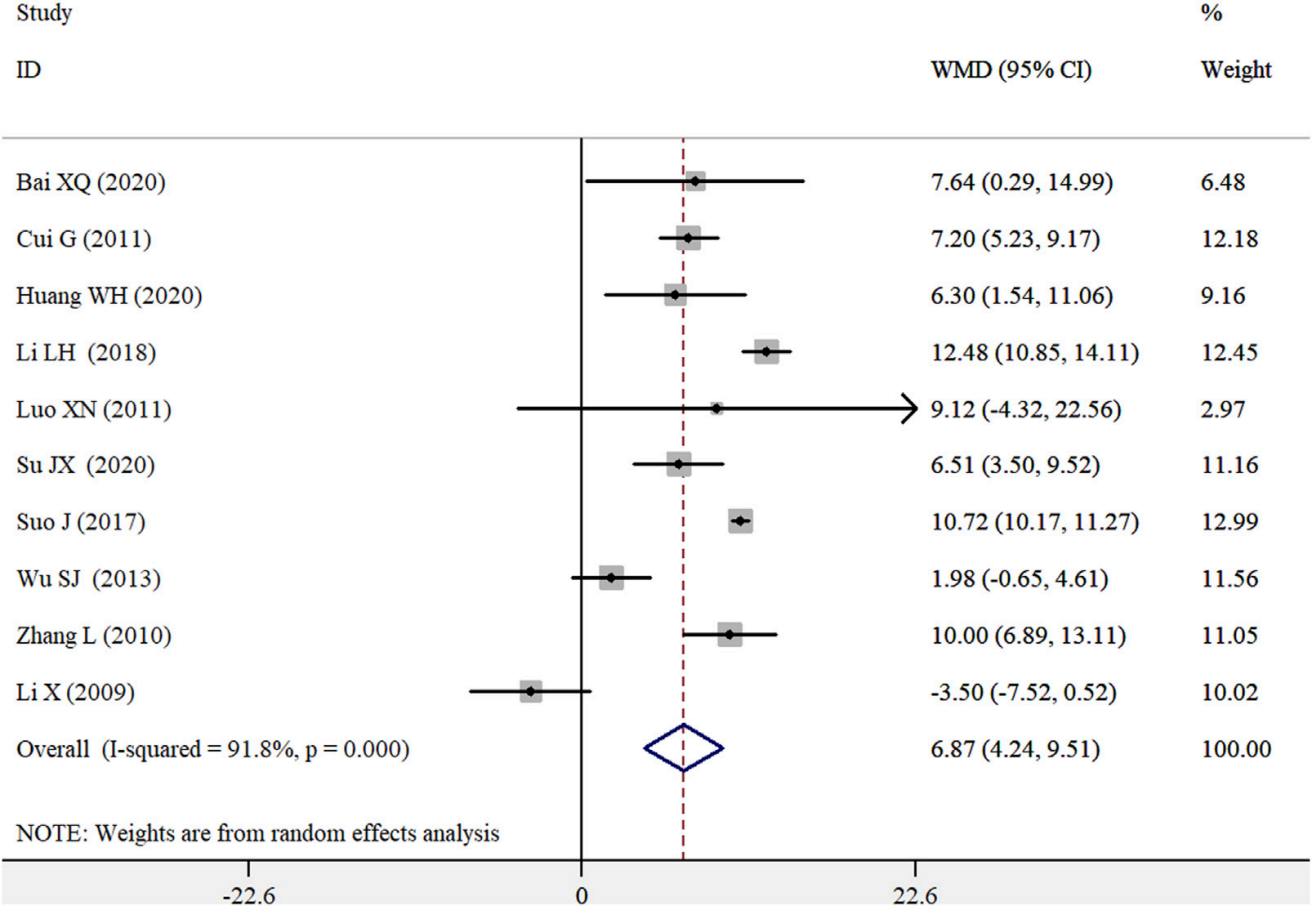
Pooled WMD with 95% CI for sperm concentration compared with or without Wu-Zi-Yan-Zong formula treatment.

Ten RCTs (Li et al., 2009; Zhang et al., 2010; Cui et al., 2011; Luo et al., 2011; Wu et al., 2013; Suo et al., 2017; Li et al., 2018; Bai et al., 2020; Huang et al., 2020; Su et al., 2020) provided data on the effect of the WZYZ formula as adjuvant therapy on sperm concentration. As illustrated in Figure 4, the pooled WMD in the sperm concentration was 6.87 × 10^6^/mL (95% CI 4.24–9.51) for the WZYZ formula combined with control therapy compared to control therapy alone, as determined by a random effects model. Significant heterogeneity was observed across the included trials (*I* ^2^ = 91.8%; *p* < 0.001). A sensitivity analysis, in which one trial was removed at a time, confirmed the reliability of the original pooled effect sizes. Except for the subgroup of varicocele/obstructive azoospermia and the WZYZ pill, the WZYZ formula as an adjuvant therapy showed a significant improvement in sperm concentration in all other subgroups (Supplementary Table S2). Egger’s test (*p* = 0.065) suggested a potential presence of publication bias, while Begg’s test (*p* = 0.721) did not. However, a trim-and-fill analysis revealed that the adjusted effect size for sperm concentration was 4.58 × 10^6^/mL (95% CI 0.26–8.91) after imputing three potentially missing trials (Supplementary Figure S3).

### 3.6 Sperm motility

**FIGURE 5:**
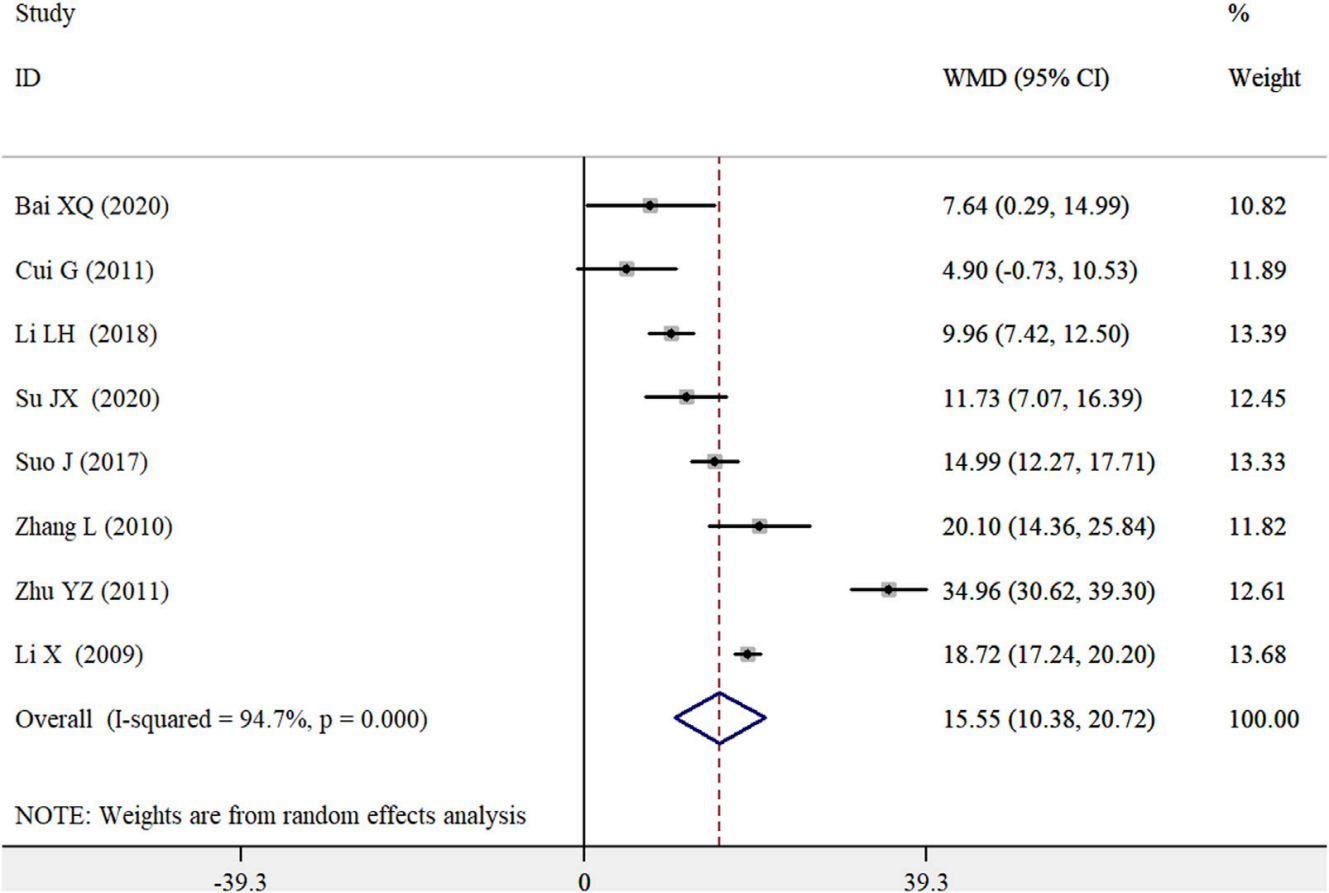
Pooled WMD with 95% CI for sperm motility compared with or without Wu-Zi-Yan-Zong formula treatment.

Eight RCTs (Li et al., 2009; Zhang et al., 2010; Cui et al., 2011; Zhu, 2011; Suo et al., 2017; Li et al., 2018; Bai et al., 2020; Su et al., 2020) examined the effects of the WZYZ formula as an adjuvant therapy on total sperm motility. As shown in Figure 5, the pooled WMD in total sperm motility was 15.55% (95% CI 10.38–20.72) for the WZYZ formula combined with control therapy compared to control therapy alone, using a random effects model. However, there was significant heterogeneity among the included studies (*I* ^2^ = 94.7%; *p* < 0.001). A leave-one-out sensitivity analysis confirmed the reliability of the original pooled effect sizes. Both Begg’s test (*p* = 0.711) and Egger’s test (*p* = 0.687) showed no evidence of publication bias.

**FIGURE 6:**
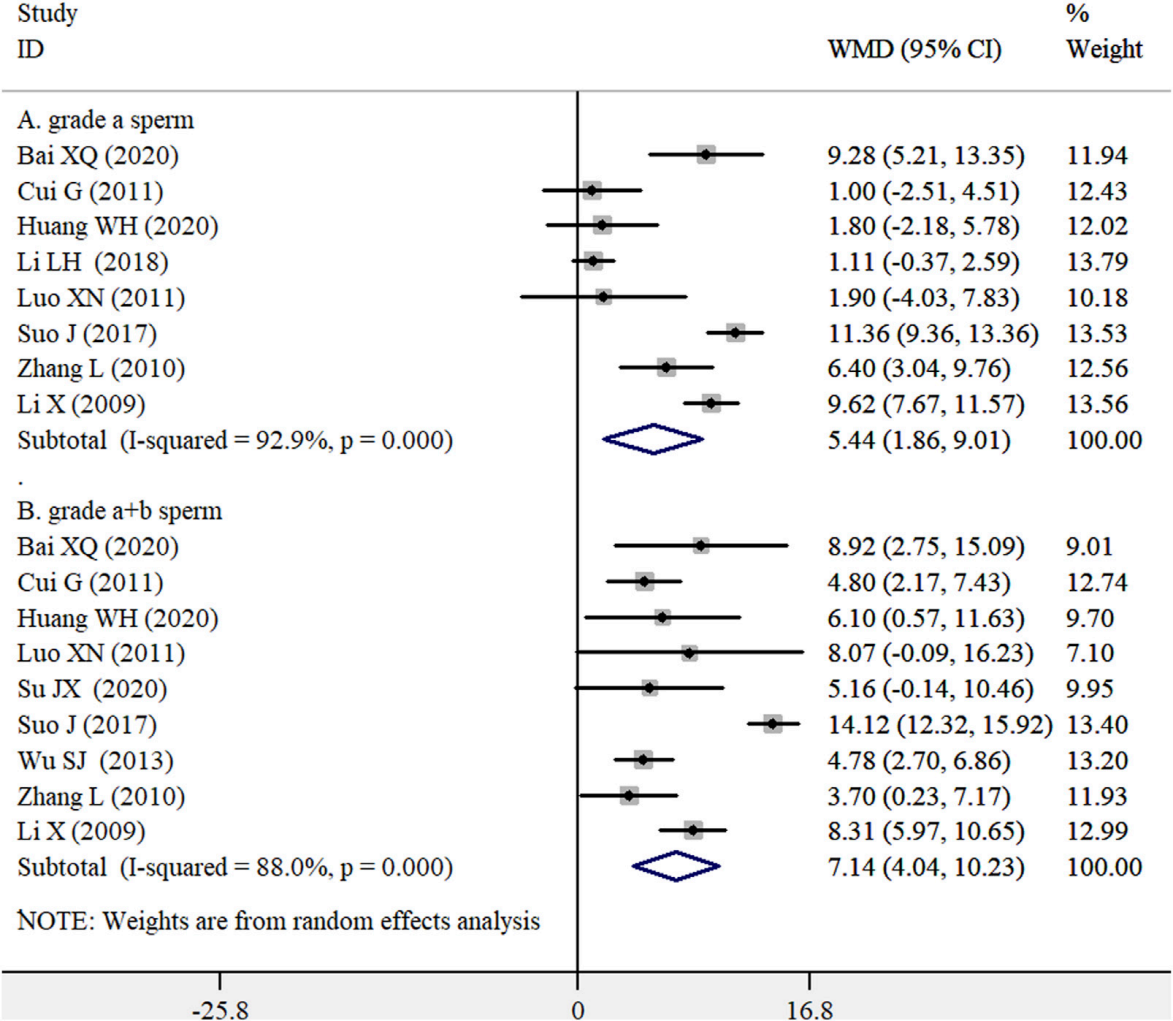
Pooled WMD with 95% CI for forward grade (a) sperm motility (A) and forward grade (a + b) sperm motility (B) compared with or without Wu-Zi-Yan-Zong formula treatment.

Eight RCTs (Li et al., 2009; Zhang et al., 2010; Cui et al., 2011; Luo et al., 2011; Suo et al., 2017; Li et al., 2018; Bai et al., 2020; Huang et al., 2020) and nine trials (Li et al., 2009; Zhang et al., 2010; Cui et al., 2011; Luo et al., 2011; Wu et al., 2013; Suo et al., 2017; Bai et al., 2020; Huang et al., 2020; Su et al., 2020) provided data on the effects of the WZYZ formula as an adjuvant therapy on forward grade (a) sperm motility and forward grade (a + b) sperm motility, respectively. As shown in Figure 6, the pooled results indicated that the combination of the WZYZ formula with control treatment significantly improved forward grade (a) sperm motility (WMD 5.44%; 95% CI 1.86–9.01; *I* ^2^ = 92.9%; *p* < 0.001) and forward grade (a + b) sperm motility (WMD 7.14%; 95% CI 4.04–10.23; *I* ^2^ = 88.0%; *p* < 0.001) compared to control treatment alone. A leave-one-out sensitivity analysis confirmed the robustness of these original pooled effect sizes. There was no evidence of publication bias for forward grade (a) sperm motility (*p* = 1.000 for the Begg’s test and *p* = 0.970 for the Egger’s test) and forward grade (a + b) sperm motility (*p* = 0.466 for the Begg’s test and *p* = 0.371 for the Egger’s test).

### 3.7 Abnormal sperm morphology and activity of acrosome enzyme

The effect of the WZYZ formula as an adjuvant therapy on abnormal sperm morphology and acrosome enzyme activity was reported in five (Luo et al., 2011; Zhu, 2011; Li et al., 2018; Huang et al., 2020; Su et al., 2020) and two (Wu et al., 2013; Bai et al., 2020) RCTs.

The pooled results, using a random effects model, showed that the combination of the WZYZ formula with the control treatment significantly reduced the percentage of abnormal sperm morphology (WMD -10.38%; 95% CI -15.72 to −5.03; *I* ^2^ = 94.3%; p < 0.001; Supplementary Figure S4) and improved the activity of the acrosome enzyme (WMD 8.02 × 10^6^ μIU; 95% CI 3.58 to 12.46; *I* ^2^ = 74.4%; p = 0.048; Supplementary Figure S5) compared to the control treatment alone. A leave-one-out sensitivity analysis demonstrated the robustness of the original pooled effect sizes for the percentage of abnormal sperm morphology (Supplementary Figure S6). Begg’s test (*p* = 1.000) and Egger’s test (*p* = 0.117) showed no evidence of publication bias for abnormal sperm morphology.

### 3.8 GRADE certainty of evidence

The certainty of the evidence is summarized in Supplementary Table S3. The overall certainty of the evidence regarding the pregnancy rate of female partners was assessed as moderate. However, the quality of evidence for semen volume, total sperm motility, forward grade (a) sperm motility, and forward grade (a + b) sperm motility was classified as low. Furthermore, the evidence for sperm concentration, abnormal sperm morphology, and acrosome enzyme activity was rated as very low quality.

## 4 Discussion

The present meta-analysis included 11 RCTs that evaluated the effectiveness of the WZYZ formula as an adjuvant therapy for treating abnormal sperm parameters associated with male infertility. The pooled results indicated that the combination of the WZYZ formula with the control treatment significantly improved the pregnancy rates of female partners and key sperm parameters, including semen volume, sperm concentration, total sperm motility, forward grade (a) sperm motility, forward grade (a + b) sperm motility, and sperm morphology, compared to the control treatment alone. These findings suggest that the WZYZ formula, when used as an adjuvant therapy, can provide additional benefits for men with abnormal sperm parameters related to infertility.

Abnormal sperm parameters are a significant contributing factor to male infertility. Common abnormalities include azoospermia (absence of sperm in the ejaculate), oligospermia (low sperm concentration), asthenospermia (reduced sperm motility), teratospermia (abnormal sperm morphology), decreased sperm viability, and elevated sperm DNA fragmentation. Addressing these abnormalities can greatly improve the chances of successful fertilization. Our meta-analysis found that adjuvant treatment with the WZYZ formula could additionally increase semen volume by 0.58 mL, sperm concentration by 6.87 × 10^6^/mL, total sperm motility by 15.55%, forward grade (a) sperm motility by 5.44%, forward grade (a + b) sperm motility by 7.14% and reduce abnormal sperm morphology by 9.35% when compared to control treatment alone. These findings suggest that the WZYZ formula, as an adjuvant therapy, has the potential to increase the likelihood of fertilization in infertile men with abnormal sperm parameters.

A significant improvement in sperm concentration, motility, or morphology may contribute to successful pregnancy. Our pooled results indicated that the WZYZ formula, when used as adjuvant therapy, could achieve a 68% increase in the pregnancy rate of female partners compared to the control treatment. The observed pregnancy rate of 35.0% following the administration of the WZYZ formula is unlikely to be attributed to chance, as evidenced by the included trials. The acrosome reaction plays a crucial role in acquiring fertilization capability (Pinto et al., 2023). Our pooled analysis demonstrated that adjuvant treatment with the WZYZ formula significantly enhanced the activity of acrosome enzymes. Male infertility can result from abnormal hormone levels, which subsequently affect sperm production. The WZYZ formula, as an adjuvant therapy, also increased serum testosterone levels but had minimal effects on follicle-stimulating hormone, prolactin, and luteinizing hormone (Cui et al., 2011; Suo et al., 2017). Furthermore, the WZYZ formula reduced sperm DNA fragmentation compared to the placebo in infertile men with DNA fragmentation exceeding 15% (Zhang et al., 2017). These findings further support the beneficial effects of the WZYZ formula on male infertility.

Varicocele is a major contributor to impaired spermatogenesis and male infertility. Approximately 40% of men with primary infertility and 12% of men with normal semen parameters are affected by varicocele (Chiba and Fujisawa, 2016). However, not all men with varicocele will experience infertility. Research has shown that varicocele repair can significantly increase the chances of pregnancy and sperm retrieval success in azoospermic men (Birowo et al., 2020). Another cause of male infertility is obstructive azoospermia, which can be effectively treated through reproductive tract reconstruction (Hayden and Tanrikut, 2015). Our subgroup analysis revealed that the WZYZ formula had a significant impact on sperm concentration and pregnancy rate in men with idiopathic asthenozoospermia, but not in cases of varicocele or obstructive asthenozoospermia. These findings suggest that the WZYZ formula may be more effective in treating idiopathic male infertility.

Regarding the side effects of the WZYZ formula, only one trial (Zhu, 2011) reported no obvious adverse events, while the remaining trials did not mention any side effects. The reported side effects of the WZYZ formula include mild gastrointestinal reactions and dizziness, which can be relieved on their own after stopping the medication (Duan and Ma, 2024). Additionally, individual allergic reactions may occur, such as skin rashes and itching. However, further in-depth studies are needed to explore specific interactions and general safety aspects of this preparation.

Diagnosis based on syndrome differentiation is a fundamental characteristic of TCM (Jiang et al., 2012). Our subgroup analysis indicated that the benefits of the WZYZ formula on pregnancy rates, sperm concentration, and total sperm motility were more pronounced in trials that categorized patients according to TCM syndrome. These findings suggest that precisely individualizing the WZYZ formula for infertile men based on TCM syndrome differentiation can enhance its efficacy. To improve the effectiveness of the WZYZ formula in addressing abnormal sperm parameters associated with male infertility, clinicians should select infertile men classified under the Kidney Yang Deficiency TCM syndrome.

This meta-analysis has several limitations that require attention. First, the quality assessment of several studies indicated an unclear risk of bias due to inadequate attention to the importance of randomization, allocation concealment, and blinding methods. These methodological flaws may have affected the certainty of the evidence. Second, the presence of significant heterogeneity in the pooled sperm parameters is another critical limitation. Variations in patient selection, specifically the lack of consideration for syndrome differentiation diagnosis, infertility etiology, preparation of the WZYZ formula, and control interventions, may have contributed to this heterogeneity. More standardized patient selection criteria, detailed reporting of infertility etiology, and consistent control interventions are necessary to address these limitations in future research. Third, Egger’s test indicated a potential presence of publication bias regarding sperm concentration outcomes. However, a trim-and-fill analysis suggested that the pooled effect size was only slightly overestimated. Fourth, the inability to assess long-term effects due to the ≤3-month treatment duration in the included studies is a notable limitation. Most of the included trials did not report long-term follow-up results. Finally, the absence of TCM syndrome differentiation during the grouping process is a significant shortcoming that has the potential to undermine the therapeutic precision of the WZYZ formula. To enhance the therapeutic precision of the WZYZ formula in future studies, it is essential to integrate TCM syndrome differentiation into the patient grouping process.

## 5 Conclusion

Adjuvant treatment with the WZYZ formula provides additional benefits in improving the pregnancy rates of female partners of infertile men with abnormal sperm parameters. The positive effects of the WZYZ formula may be associated with enhancements in semen parameters, including semen volume, sperm concentration, motility, and morphology. However, further well-designed RCTs with larger sample sizes are essential to definitively establish the efficacy and safety of the WZYZ formula in addressing abnormal sperm parameters related to male infertility.

## Data Availability

The original contributions presented in the study are included in the article/Supplementary Material, further inquiries can be directed to the corresponding author.
