# Supplementary material for: Adjuvant treatment with Wu-Zi-Yan-Zong formula for abnormal sperm parameters associated with male infertility: a meta-analysis of randomized controlled trials
Source: Front Pharmacol. 2025 May 6;16:1580705. doi: 10.3389/fphar.2025.1580705 (PMC12089090; doi:10.3389/fphar.2025.1580705)
Supplement: Supplementary file 1 [file Table1.doc]

~~Table S1 Results of subgroup analysis on pregnancy rate of female partners~~

| Subgroups | Number of trials | Pooled RR | 95% CI | Heterogeneity between studies |
| --- | --- | --- | --- | --- |
| Grouping based on TCM syndrome  Yes  No | 3  6 | 1.74  1.64 | 1.22 to 2.46  1.21 to 2.22 | *p*=0.984; *I*^2^ = 0.0%  *p*=0.910; *I*^2^ = 0.0% |
| Form of WZYZ  Pill  Capsule | 6  3 | 1.61  1.79 | 1.21 to 2.14  1.23 to 2.60 | *p*=0.937; *I*^2^ = 0.0%  *p*=0.941; *I*^2^ = 0.0% |
| Etiology of infertility  Varicocele/Obstructive  Idiopathic | 2  7 | 1.35  1.77 | 0.89 to 2.05  1.36 to 2.31 | *p*=0.998; *I*^2^ = 0.0%  *p*=0.997; *I*^2^ = 0.0% |

TCM, traditional Chinese medicine; WZYZ, Wu-Zi-Yan-Zong; RR, risk ratio; CI, confidence interval
