# Supplementary material for: Adjuvant treatment with Wu-Zi-Yan-Zong formula for abnormal sperm parameters associated with male infertility: a meta-analysis of randomized controlled trials
Source: Front Pharmacol. 2025 May 6;16:1580705. doi: 10.3389/fphar.2025.1580705 (PMC12089090; doi:10.3389/fphar.2025.1580705)
Supplement: Supplementary file 2 [file Table3.docx]

Table S3 GRADE of evidence of outcomes of the included trials

| Outcome measures |  | Certainty assessment | | | | | Effect | | Certainty | Importance |
| --- | --- | --- | --- | --- | --- | --- | --- | --- | --- | --- |
|  | No. of trials | Risk of bias | Inconsistency | Indirectness | Imprecision | Publication bias | No. of patients | Effect sizes |  |  |
| Pregnancy rate of female partners | 9 | Serious | Not serious | Not serious | Not serious | No | 761 | RR 1.68 (95%CI 1.34 to 2.11) | ⊕⊕⊕⊝ **Moderate** | Critical |
| Semen volume | 6 | Serious | Serious | Not serious | Not serious | No | 594 | WMD 0.58 mL (95% CI 0.28 to 0.89) | ⊕⊕⊝⊝ **Low** | Important |
| Sperm concentration | 10 | Serious | Serious | Serious | Not serious | Likelihood# | 851 | WMD 6.87 ×10^6/ml (95% CI 4.24 to 9.51) | ⊕⊝⊝⊝ **Very low** | Critical |
| Total sperm motility | 8 | Serious | Serious | Not serious | Not serious | No | 837 | WMD 15.55% (95% CI 10.38 to 20.72) | ⊕⊕⊝⊝ **Low** | Critical |
| Forward sperm motility (grade a) | 8 | Serious | Serious | Not serious | Not serious | No | 690 | WMD 5.44% (95% CI 1.86 to 9.01) | ⊕⊕⊝⊝ **Low** | Critical |
| Forward sperm motility (grade a + b) | 9 | Serious | Serious | Not serious | Not serious | No | 731 | WMD 7.14% (95% CI 4.04-10.23) | ⊕⊕⊝⊝ **Low** | Critical |
| Percentage of abnormal morphology | 5 | Serious | Serious | Not serious | Serious | No | 434 | WMD -10.38% (95% CI -15.72 to -5.03) | ⊕⊝⊝⊝ **Very low** | Important |
| Sperm acrosome enzyme | 2 | Serious | Serious | Not serious | Serious | Undetected | 138 | WMD 8.02 × 10^6 μIU (95% CI 3.58 to 12.46) | ⊕⊝⊝⊝ **Very low** | Important |

WMD, weighted mean difference; RR, risk ratio; CI, confidence interval

Downgraded by one level for following reason: Risk of bias; serious, study with unclear risk of bias; Inconsistency: Serious, *I*^2^>50%. Indirectness of evidence, no indirectness of evidence was observed in any trial. Imprecision: Serious, sample size less than 500 patients. Publication bias: Egger’s test indicated the likelihood of publication bias and trim-and-fill analysis did change the statistical significance.
