# Supplementary material for: Adjuvant treatment with Wu-Zi-Yan-Zong formula for abnormal sperm parameters associated with male infertility: a meta-analysis of randomized controlled trials
Source: Front Pharmacol. 2025 May 6;16:1580705. doi: 10.3389/fphar.2025.1580705 (PMC12089090; doi:10.3389/fphar.2025.1580705)
Supplement: Supplementary file 3 [file DataSheet2.docx]

**Supplementary Text S1 Search strategy**

| **1. PubMed** | Query | | Items found |
| --- | --- | --- | --- |
| #1 | Search: **(Wuzi Yanzong) OR (Wu zi Yan zong)** | | 79 |
| #2 | Search: **((((male infertility) OR (sperm)) OR (oligospermia)) OR (asthenospermia)) OR (oligoasthenospermia)** | | 167,603 |
| #3 | Search: **(#1) AND (#2)** | | **37** |
|  |  | |  |
| **2. Embase** | Query | Items found | |
| #1 | Search 'wuzi yanzong'/exp OR 'wuzi yanzong' OR (wuzi AND yanzong) | 115 | |
| #2 | Search 'wu zi yan zong' OR (wu AND zi AND yan AND zong) | 50 | |
| #3 | Search #1 OR #2 | 165 | |
| #4 | Search 'male infertility'/exp OR 'male infertility' OR (('male'/exp OR male) AND ('infertility'/exp OR infertility)) | 96,658 | |
| #5 | Search 'sperm'/exp OR sperm | 178,149 | |
| #6 | Search 'oligospermia'/exp OR oligospermia | 9,946 | |
| #7 | Search 'asthenospermia'/exp OR asthenospermia | 3,493 | |
| #8 | Search 'oligoasthenospermia'/exp OR oligoasthenospermia | 9,377 | |
| #9 | Search #4 OR #5 OR #6 OR #7 OR #8 | 226,499 | |
| #10 | Search #3 AND # 9 | **49** | |

**3. Cochrane Library**

Search: wuzi yanzong in Title Abstract Keyword OR wu zi yan zong in Title Abstract Keyword  **34 items**

| **4.** **Web of Science** | Query | Items found |
| --- | --- | --- |
| #1 | Search **wuzi yanzong**(Topic) or **wu zi yan zong** (Topic) | 109 |
| #2 | Search **male infertility (Topic) or sperm (Topic) or oligospermia (Topic) or asthenospermia (Topic) or oligoasthenospermia** (Topic) | 222,889 |
| #3 | Search **#1 AND #2** | **43** |

**5.** **China National Knowledge Infrastructure**

Search (主题: 男性不育) OR（篇关摘：精子）AND（篇关摘：五子衍宗）AND (全文：随机) AND (全文：对照) **278 items**

**6. Wanfang Databases**

Search ((((主题="男性不育") OR "精子") AND 题名或关键词="五子衍宗") AND "随机") AND "对照" **98 Items**

**7. Chinese Biomedical Literature Database (Sinomed)**

Search ("男性不育"[全部字段] OR "精子"[全部字段]) AND "五子衍宗"[常用字段] **314 items**
