# Supplementary material for: Adjuvant treatment with Wu-Zi-Yan-Zong formula for abnormal sperm parameters associated with male infertility: a meta-analysis of randomized controlled trials
Source: Front Pharmacol. 2025 May 6;16:1580705. doi: 10.3389/fphar.2025.1580705 (PMC12089090; doi:10.3389/fphar.2025.1580705)

Supplemental Figure S1. Results of the leave-one-out sensitivity analysis on the pregnancy rate of female partners.


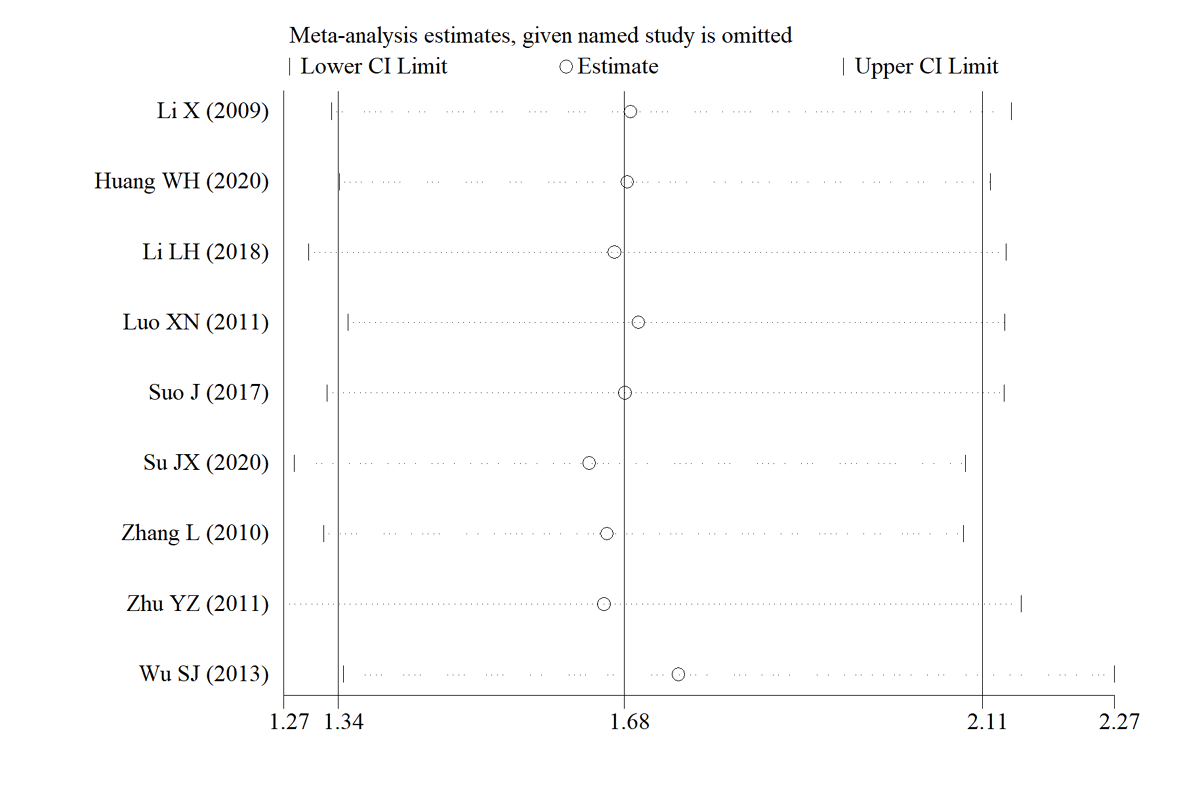


Figure S2. Pooled WMD with 95% CI of semen volume compared treatment with and without the Wu-Zi-Yan-Zong formula.


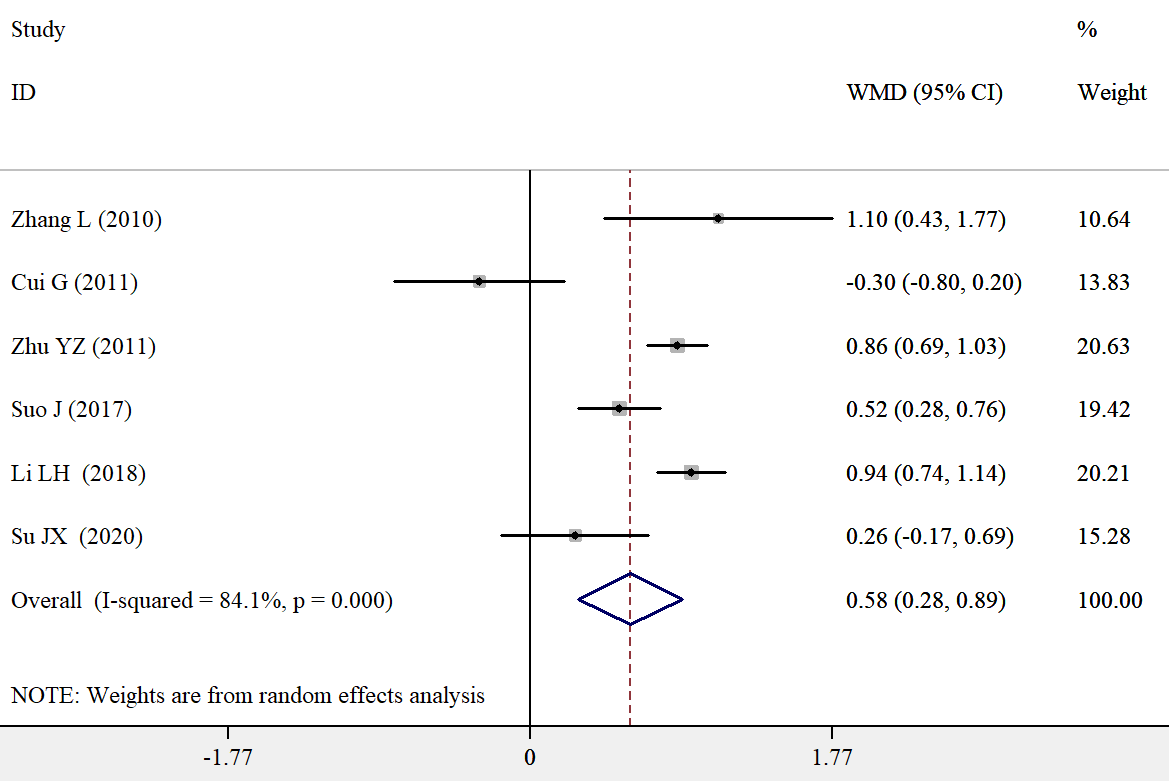


Supplemental Figure S3. The trim-and-fill analysis shows the adjusted effect of WZYZ formula as an adjuvant therapy on sperm concentration. The circles alone are real studies, while the circles enclosed in boxes are ‘filled’ studies.


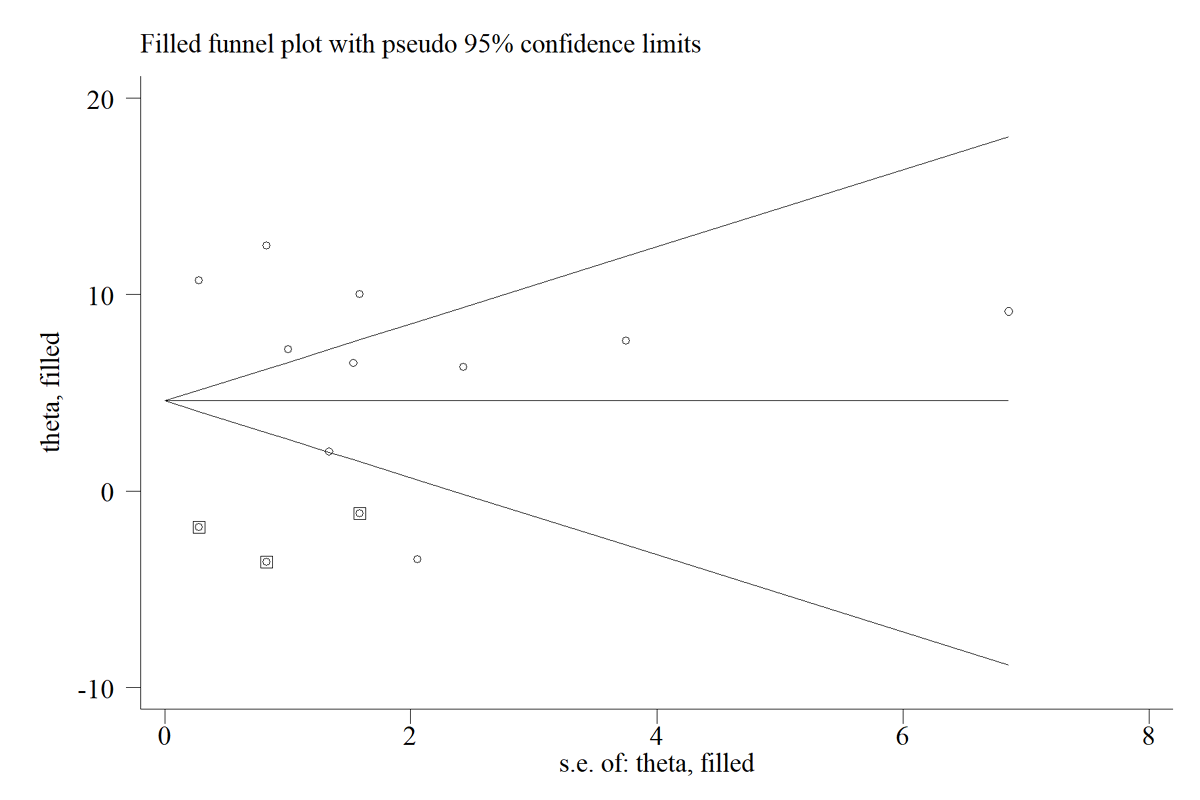


Figure S4. Pooled WMD with 95% CI of the abnormal sperm morphology compared treatment with and without the Wu-Zi-Yan-Zong formula.


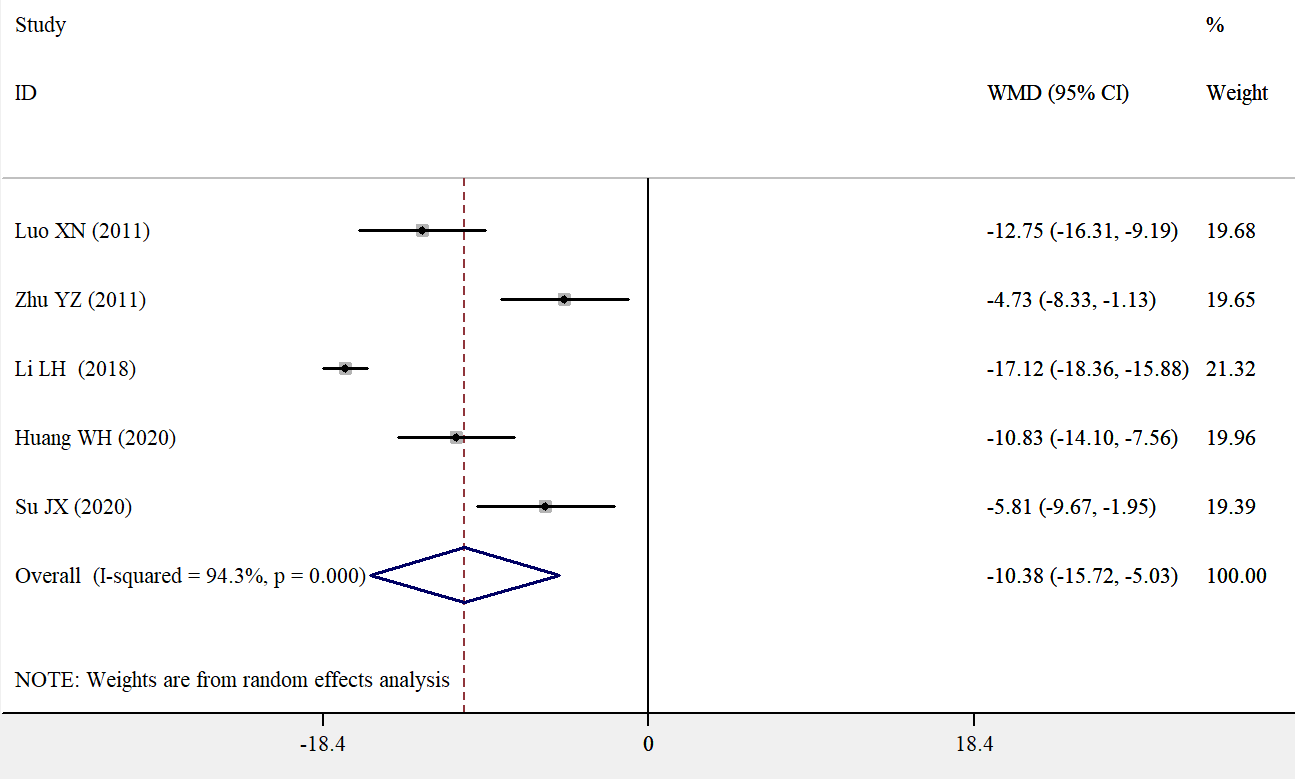


Figure S5. Pooled WMD with 95% CI of acrosome enzymeactivity compared treatment with and without the Wu-Zi-Yan-Zong formula.


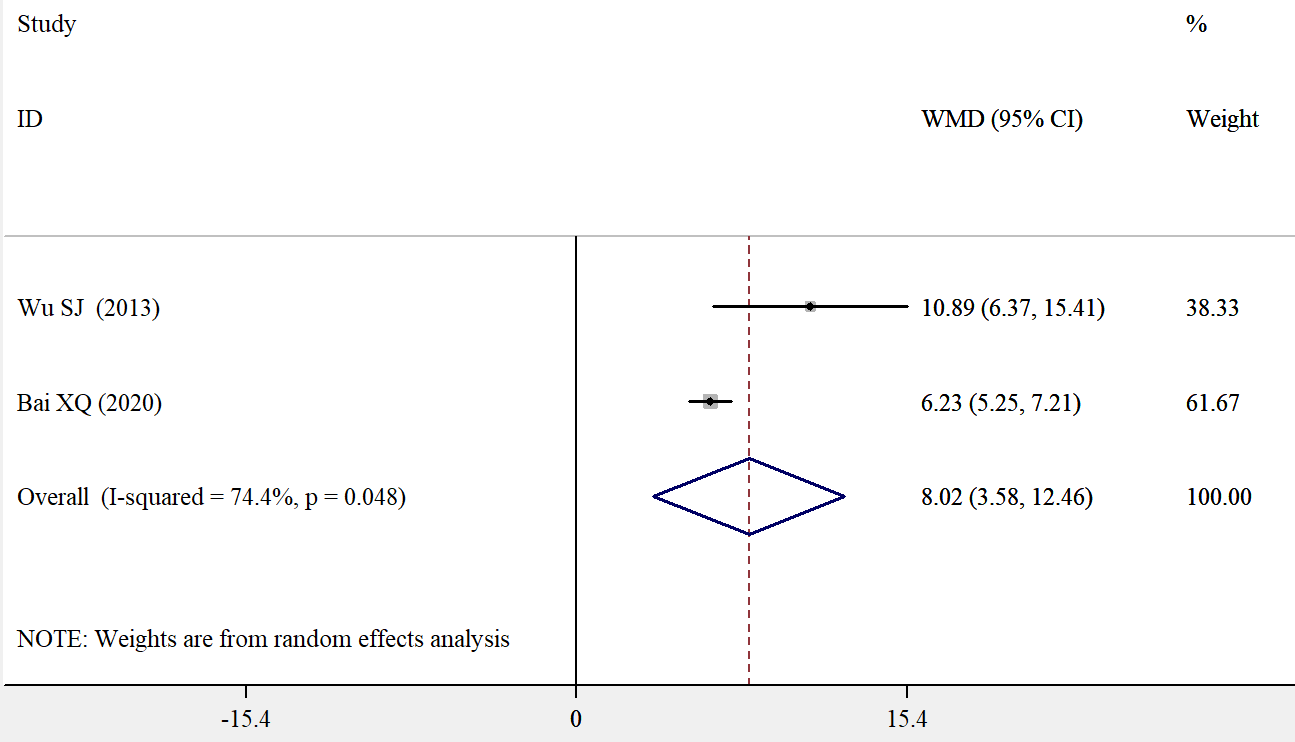


Supplemental Figure S6. Results of the leave-one-out sensitivity analysis on the percentage of abnormal sperm morphology.


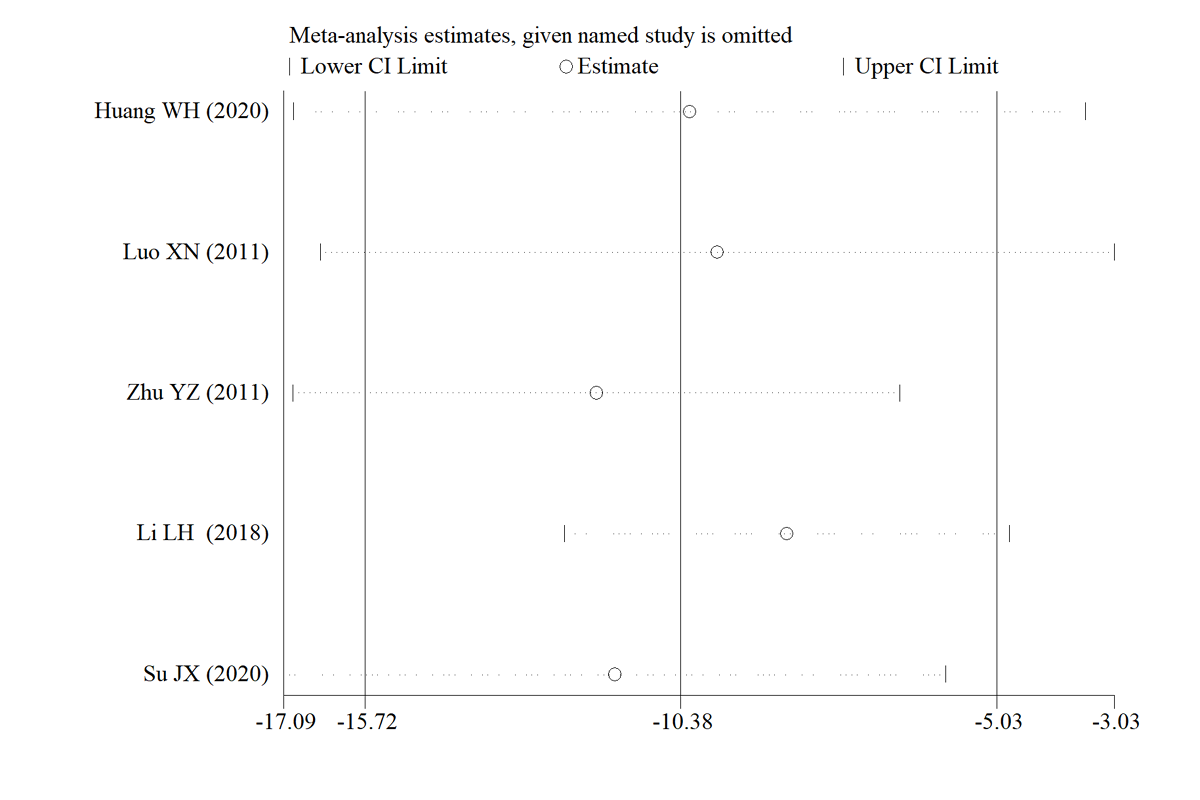

Supplement: Supplementary file 4 [file DataSheet1.docx]
