# Supplementary material for: Adjuvant treatment with Wu-Zi-Yan-Zong formula for abnormal sperm parameters associated with male infertility: a meta-analysis of randomized controlled trials
Source: Front Pharmacol. 2025 May 6;16:1580705. doi: 10.3389/fphar.2025.1580705 (PMC12089090; doi:10.3389/fphar.2025.1580705)
Supplement: Supplementary file 5 [file Table2.doc]

~~Table S2 Results of subgroup analysis on sperm concentration~~

| Subgroups | Number of trials | Pooled WMD (× 10^6^/ml) | 95% CI | Heterogeneity between studies |
| --- | --- | --- | --- | --- |
| Grouping based on TCM syndrome  Yes  No | 2  8 | 9.81  6.03 | 3.81 to 15.81  2.63 to 9.43 | *p*=0.016; *I*^2^ = 82.7%  *p*<0.001; *I*^2^ = 92.9% |
| Form of WZYZ  Pill  Capsule  Decoction | 5  3  2 | 4.23  10.33  7.23 | -1.00 to 9.45  8.12 to 12.54  5.33 to 9.13 | *p*<0.001; *I*^2^ = 87.2%  *p*=0.003; *I*^2^ = 83.3%  *p*=0.910; *I*^2^ = 0.0% |
| Etiology of infertility  Idiopathic  Varicocele/Obstructive | 8  2 | 7.55  2.38 | 5.02 to 10.07  -0.84 to 5.60 | *p*<0.001; *I^2^* = 90.4%  *p*=0.307; *I*^2^ = 4.2% |

TCM, traditional Chinese medicine; WZYZ, Wu-Zi-Yan-Zong; WMD, weighted mean difference; CI, confidence interval.
